# CAR-T cell therapies are coming after glioblastoma: An overview of early phase clinical trials and future perspectives

**DOI:** 10.1016/j.isci.2025.114609

**Published:** 2026-01-02

**Authors:** Annapaola Mariniello, Denis Migliorini

**Affiliations:** 1 Department of Oncology, University Hospital of Geneva, Geneva, Switzerland; 2 Department of Pathology and Immunology, University of Geneva, Geneva, Switzerland; 3 Center for Translational Research in Onco-Hematology, University of Geneva, Geneva, Switzerland; 4 AGORA Cancer Research Center, 1005 Lausanne, Switzerland; 5 Swiss Cancer Center Léman, Geneva, Lausanne, Switzerland

**Keywords:** Immunology, Oncology, Therapeutics

## Abstract

Recurrent glioblastoma has no established standard of care, and survival remains limited to a few months. Recent clinical evidence suggests that the intracranial delivery of CAR-T cells is feasible and shows signs of antitumor activity. In the phase I trial NCT02208362, anti-IL13Rα2 CAR-T cells were tested in 65 patients, yielding a 50% disease-control rate and 23% one-year survival. Two phase I studies of multi-target CAR-T products (NCT05168423, the ongoing NCT05660369) similarly showed bioactivity and early but transient radiological responses. Importantly, the intracranial route enabled rich translational research: serial biopsies and cerebrospinal fluid sampling allowed assessment of CAR-T cells function at the tumor site and the identification of candidate predictive biomarkers. In this review, we compare the designs and findings of these three studies and synthesize key lessons. We also outline future perspectives to improve the efficacy of CAR-T cell therapy in solid tumors, with a focus on glioblastoma.

## Graphical abstract

**Figure:**
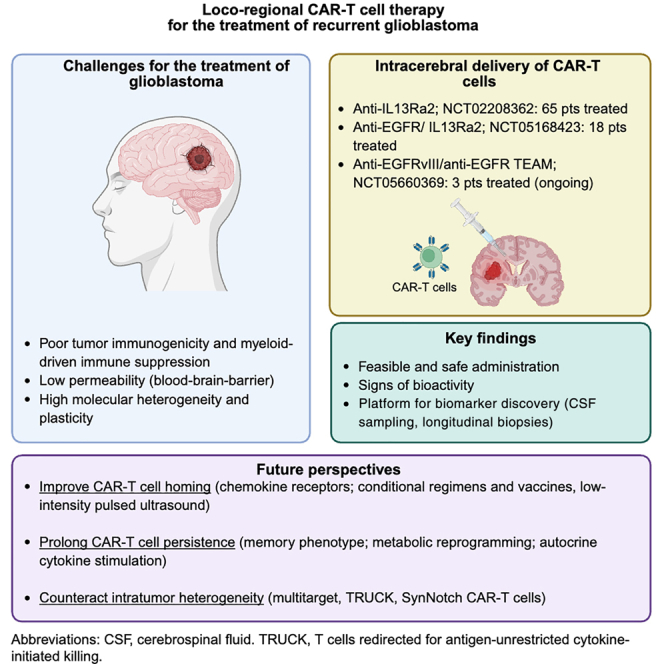

## Glioblastoma: A death sentence for patients

Glioblastoma (GBM) is the most common and malignant primary brain tumor of the adult, with an incidence of 3.2 per 100,000 cases/year in the United States and a 5-year survival rate <10%.^1^ Histologically, it is classified as a World Health Organization grade 4 glioma and is typically characterized by pseudopalisading necrosis, microvascular proliferation, and anaplasia.^2^^,^^3^

While GBM is conventionally an astrocytic tumor, there is ongoing debate on the true cell of origin of GBM. New evidence propose that neural stem cells in the subventricular zone give rise to precancerous cells that ultimately evolve in heterogeneous tumors by activating multiple programs crucial for gliomagenesis.^4^^,^^5^ The molecular signature of GBM largely varies, yet the absence of genetic mutations in the isocitrate dehydrogenase (IDH 1–2) and H3 genes discriminates GBM from grade 4 gliomas evolved from lower-grade forms.^2^^,^^6^ As seizure and cognitive disorder are the most common clinical presentation, GBM carries a high social and psychological burden on patients and caregivers.

The standard-of-care therapy for GBM consists of tumor resection, followed by concomitant chemo-radiotherapy and consolidation chemotherapy based on temozolomide.^2^^,^^7^ This standard has remained unchanged for almost two decades and, only recently, it has been combined with tumor-treating fields, given in the consolidation phase.^8^ For treated patients, prognosis is 15–17 months and also depends on the MGMT methylation status, which, when positive (45% of the cases), is associated with response to temozolomide and to other alkylating agents.^2^^,^^8^ Dramatically, at the time of relapse after this multi-modality treatment (usually less than 7 months), no treatment strategy provides significant survival benefit over supportive care.^2^

In this context, the identification of effective treatment strategies ameliorating survival time and quality of life for patients with recurrent GBM is a research priority. So far, multiple approaches have been tested in recurrent GBM, spanning from targeted therapy to diverse immunotherapies, all of which failed to prove effective, likely as a result of intrinsic biologic limitations. First of all, as for all brain tumors, the blood-brain barrier (BBB) is responsible for the low permeability of monoclonal antibodies and for acquired or primary resistance to chemotherapy.^9^ Second, GBM is an immunologically “cold tumor” characterized by a low tumor mutational burden and a highly immunosuppressive microenvironment, rich of hypoxic and necrotic regions, which make it an inhospitable environment for infiltration of effector T cells.^10^^,^^11^^,^^12^ The microenvironment of the central nervous system is evolutionarily conserved to protect the vital neurologic function from inflammation and explains why GBM is refractory to immunotherapy with PD-1 targeted strategies,^13^^,^^14^ even in the presence of common predictors of response to checkpoint inhibitors, like high PD-L1.^15^^,^^16^^,^^17^

One more challenge to the successful treatment of GBM is the high plasticity and the molecular heterogeneity, with a rich compensatory signaling network. The absence of stable oncogenic drivers limits the development of effective targeted therapies in GBM.^18^^,^^19^^,^^20^ About 25–30% of all patients with GBM express the EGFRvIII neoantigen, which is an EGFR variant resulting from the in-frame deletion of exons 2–7.^21^ Therapeutic strategies targeting EGFRvIII included the peptide vaccine rindopepimut^22^ and the bispecific antibody depatuxizumab mafodotin^23^, both yielding negative results.

## CAR-T cell therapy for glioblastoma

The poor immunogenicity and the hostile microenvironment for endogenous T cell functionality make GBM a good candidate for engineered immunotherapy with chimeric-antigen receptor T cells (CAR-T). These T cells are manipulated for the replacement of the T cell receptor (TCR) with a modified immunoglobulin (scFv), composed of a heavy chain and a light chain, directed against an epitope of interest.^24^^,^^25^ Upon binding to this target epitope exposed on cancer cells, the engagement of the scFv will elicit an intracellular signaling, stimulatings T cell activation thus resulting in direct cytotoxicity, production of cytokines, and CAR-T cell expansion.^24^^,^^26^ As opposed to immunotherapy with checkpoint blockade, the anti-tumor activity of CAR-T therapy does not rely on the reinvigoration of a pre-existing adaptive immunity but is capable of direct and specific granzyme/perforin-mediated cytotoxicity, independently from the antigen-presentation machinery and extrinsic co-stimulatory signals.^27^^,^^28^ To enhance the expansion potential and the *in vivo* lifespan, the intracellular domain of CAR-T has evolved through four generations, from CD3ζ as the only downstream signal, as in native TCR, to the incorporation of one or more co-stimulatory molecules like CD28, 41-BB, or OX-40 (second and third generation), to the latest generation of CAR-T, additionally modified with a constitutively expressed or inducible transgenic protein which is usually a cytokine.^25^^,^^26^

### Challenges of CAR-T cells in solid tumors

Despite significant technical advancements, the use of CAR-T cell for the treatment of solid malignancies faces several limitations. First, the low intra-tumor penetration of CAR-T cells is one of the main reasons why the success in hematological malignancies has not been recapitulated yet in solid tumors.^29^ Indeed, the dense tumor stroma, the hypoxia resulting from the disrupted neovascularization, and the presence of immune-suppressive cells (microglia, neutrophils) all represent a barrier for T cell trafficking and persistence.^29^^,^^30^ To improve the survival and penetration of CAR-T cells in GBM, loco-regional approaches were developed through intratumoral, intraventricular, or intracranial injection. This route of administration for CAR-T cells is particularly challenging, as the inflammation-induced edema can be potentially severe, with increased intracranial pressure. The future perspectives to improve CAR-T cells homing in GBM are discussed in section “Perspectives to improve CAR-T cell homing at the tumor site”.

Second, when considering the whole process preceding CAR-T cells administration, including leukapheresis, potential conditional chemotherapy, T cell isolation, expansion, and lentiviral CAR transduction, the turnaround time can be long and costly.^31^ This latter aspect, in particular, is considered a major constraint for large-scale applicability of adoptive cell therapies.

Third, the side effects of CAR-T cell therapy can be life-threatening or invalidating and, so far, difficult to prevent.^32^ CAR-T cell toxicity mostly consists of a cytokine release syndrome (CRS), indicating an acute systemic inflammatory syndrome that results from an uncontrolled and dysregulated immune response, where the CAR-T cell releases IL-6, IFN-γ, IL-1, and IL-2 and activates the downstream myeloid cells with the further release of inflammatory mediators.^32^ Moderate/severe CRS can be often associated with a neurologic condition defined as immune effector cells associated neurotoxicity syndrome (ICANS), where the local and systemic production of IL-6 and IL-1 seem to be involved^33^ and it occurs concomitantly or a few days after CRS.^32^ Interestingly, ICANS more often occurs in CAR-T products containing the CD28 intracellular domain, as this is able to elicit an intense CAR-T cell proliferation and cytokine production.^34^ In some cases, immune-mediated neurotoxicity may also result from on-target, off-tumor effects. This could be the case of CAR-T cells targeting CD19, as mural cells lining the BBB also express CD19.^35^ Recently, CAR-T cells-induced hemophagocytic disorders have also been described as a severe complication of CRS, even though the underlying mechanisms are still unknown.^36^^,^^37^ Less characterized, longer-term side effects of CAR-T cell therapy include increased vulnerability to infections, immune effector cell-associated myelotoxicity, or the onset of secondary malignancies, usually T cell lymphoproliferative disorders, which is still extremely rare.^32^ Treatment for CAR-T cell toxicity consists of immune-suppressive agents, where the anti-IL-6R tocilizumab is usually the first-line treatment for CRS, while high-dose steroids are front-line in case of ICANS. Anti-IL1R or JAK inhibitors are also employed, even though their role is less established.^32^

Lastly, critical challenges of CAR-T cell therapy are the short persistence of CAR-T in the tumor and blood and the development of acquired resistance, due to CAR-T cell exhaustion and in particular to the downregulation of the tumor-associated CAR-T cell targets. Similarly to native CD8 T cells, CAR-T cell exhaustion indicates a progressive loss of cytotoxic and proliferative abilities induced by tonic intracellular signaling upon antigen binding and is characterized by the upregulation of the co-inhibitory receptors PD-1, TIM-3, CTLA-4, and LAG-3, among others.^38^ This phenomenon has been observed in CAR-T cells used for the treatment of both hematologic and solid tumors, and currently, T cell engineering approaches are underway to overcoming this acquired dysfunction, as discussed in section “new synthetic T cell constructs”. As for immune escape and tumor heterogeneity, in GBM, downregulation of the CAR-T target antigens EGFRvIII and IL13Rα2 has been well documented comparing pre- and post-treatment tumor biopsies, and it seems to occur early.^39^^,^^40^ The most promising research efforts to tackle this challenge are discussed in section “perspectives to circumvent tumor heterogeneity and immune escape.”

### Previous clinical experience of CAR-T cells in glioblastoma

The key data from the pilot studies of CAR-T cell therapy in GBM are summarized in Table 1. The first-in-human CAR-T cell therapy for GBM consisted in the intracranial administration of a first-generation T cell product targeting interleukin 13 receptor subunit alpha 2 (IL13Rα2), called IL-13 zetakine.^40^ IL13Rα2 is a cancer testis antigen present in about 75% of GBM, with limited expression in healthy tissues^49^ and it is a marker of poor prognosis.^50^^,^^51^ In the first report published in 2015,^40^ 3 patients with recurrent GBM received up to 4 anti-IL13Rα2 CAR-T cell injections (average dose of 10^8^) in the resection cavity after gross tumor removal. Side effects, mostly neurological, were transient and manageable. Clinical activity was observed in 2 of 3 patients. A case report followed, documenting a complete response after multiple doses of intra-cavity and intra-ventricular CAR-T cells.^41^ In this case, an improved version of the IL-13 zetakine was used, consisting of a second-generation construct with integration of a 4-1BB intracellular domain, and of a mutated IgG4-Fc linker to reduce interaction with Fc-expressing myeloid cells. In addition, this IL13BBζ CAR was transduced in T cells enriched for a central memory phenotype. In a separate phase I trial,^42^ an allogeneic version of the first-generation IL-13 zetakine was tested in recurrent GBM. This CAR-T product was engineered to be refractory to glucocorticoids and injected intracranially in combination with a recombinant IL-2 in 4 doses. Transient clinical activity was observed but CAR-T cells showed short intra-tumor persistence, where the authors proposed as potential causes: allo-rejection of CAR-T cells (anti-CAR T cells antibodies found at least in one case), detrimental effect of dexamethasone on activated endogenous immune cells, intrinsic lower potency of the first-generation CAR-T cell product, and excessively long *ex vivo* expansion time.

**Table 1:** Summary of pilot/phase I clinical trials of CAR-T cell therapy in glioblastoma published before March 2024

Target Antigen	CAR Generation/Construct	Administration Route	Trial Phase/Design	#Cases	Main Outcomes	Trial Identifier	Reference
IL13Rα2	1st-generation IL-13 zetakine	Intracavitary (resection cavity)	Pilot/first in human	3	Manageable transient neurological adverse events; clinical activity in 2/3 patients	NCT02208362	^40^
	2nd-generation IL13BBζ with 4-1BB and mutated IgG4-Fc linker, CM-enriched T cells	Intraventricular + intracavitary	Case report	1	Complete response lasting 7.5 months	Not Available	^41^
	1st-generation glucocorticoid-resistant CAR, allogenic	Intracavitary + rIL-2	Phase I	6	Transient activity (4/6); limited persistence (allo-rejection, steroid effect)	Not Available	^42^
EGFRvIII	2nd-generation CAR (CD28.ζ)	Intravenous	Pilot	10	Transient response in 1 pt; antigen loss and adaptive resistance	NCT02209376	^39^
	2nd-generation CAR + lymphodepletion ± IL-2	Intravenous	Phase I	18	3 pts survived >1 year; no objective responses; one treatment-related death	Not Available	^43^
	Anti-EGFRvIII CAR-T + pembrolizumab	Intravenous (×3 infusions)	Phase I	7	Well tolerated; no responses; minimal CAR-T expansion/persistence	NCT03726515	^44^
HER2	2nd-generation CD28.ζ virus-specific T cells co-expressing HER2 CAR	Intravenous	Phase I dose escalation	17	Disease control 8/17; 1 partial response; persistence up to 12 months; no *in vivo* expansion	NCT01109095	^45^
EphA2	2nd-generation CAR (costimulatory not specified)	Intravenous	First in human (3 patients)	3	Safe; transient tumor regression in 1 case	Not Available	^46^
B7-H3	4-1BBζ CAR-T	Intravenous/regional	Early phase/case report	1	Radiographic regression	NCT04077866; NCT05366179	^47^
GD2	4SCAR-T (4-signal self-co-stimulating construct)	Intravenous	Phase I	14	Manageable toxicity; preliminary anti-tumor activity	Not Available	^48^

The EGFRvIII antigen is the most common type of EGFR mutation, and it is present in half of the cases of EGFR overexpression.^52^ Its expression is strongly tumor-restricted, making it a valuable CAR-T cell target. CAR-T cells targeting EGFRvIII were tested intravenously in a small trial,^39^ where only one patient reported a durable progression-free survival (PFS). Interestingly, 7 patients underwent surgery after CAR-T cell infusion (mostly within one month), allowing the pre- and post-treatment analysis of the tumor. While CAR-T cells expanded in the blood and infiltrated the tumor, upregulation of immune-suppressive molecules (PD-L1, IDO-1) and accumulation of T-regulatory cells were detected, as well as the downregulation of EGFRvIII. To increase the persistence of CAR-T cells, an ensuing anti-EGFRvIII CAR-T cell phase I trial attempted conditional lymphodepleting chemotherapy and sequential IL-2 administration. Three of the 18 patients survived over one year, despite the absence of objective responses.^43^ Safety was a critical issue, with a toxic death from pulmonary edema and hypotension. More recently, anti-EGFRvIII CAR-T cells were combined to PD-1 blockade in 7 patients with newly diagnosed MGMT unmethylated GBM.^44^ After a short-course radiation therapy, 3 doses of anti-EGFRvIII CAR-T cells and pembrolizumab were followed by a 4^th^ dose of pembrolizumab alone. Treatment was well tolerated, but no sign of clinical activity was noted. Peripheral expansion and persistence of CAR-T was minimal, despite the repeated infusions. Analysis in post-treatment tumor tissue showed limited CAR-T cell intra-tumor infiltration. Of note, intra-tumor activation of the myeloid and T cell compartments was also found, with bystander T cells seeming to persist in the tumor longer than CAR-T cells. Additionally, increased interferon (IFN)-stimulated T cells were favorably associated with post-progression survival time.

HER2 is another target employed for CAR-T cell therapy in GBM. Anti-HER2 CAR-T cells were administered peripherally in relapsed GBM in a phase I trial.^45^ With the aim of augmenting the antigenicity and the endogenous immune response, second-generation (CD28.ζ) engineered T cells were first transduced with a dominant cytomegalovirus epitope, and then with the anti-HER2 scFv. Disease control rate was obtained in 8 of 17 cases, including one long-term partial response, in the absence of dose-limiting toxicities. Even though CAR-T cells persisted in the blood up to 12 months, they did not expand *in vivo* after infusion, suggesting early exhaustion after *in vitro* antigen stimulation and culture.

Other targets have been investigated for CAR-T cell therapy of GBM, including EphA2, B7-H3, and disialoganglioside 2 (GD2), tested in early-phase/pilot clinical trials, overall with modest results.^45^^,^^46^^,^^47^^,^^48^

## Novel clinical evidence on the loco-regional administration of CAR-T cells

In March 2024, three phase I trials testing loco-regional administration of CAR-T cells confirmed the feasibility of the loco-regional approach and showed a transient anti-tumor activity. Here, we compare and contrast the clinical and translational findings of the three studies (Table 2).

**Table 2:** Comparative summary of the phase I trials testing loco-regional administration of CAR-T for recurrent glioblastoma published since March 2024

		Anti-IL13Rα2 CAR-T (Brown et al.)	Anti-EGFR/IL13Rα2 CAR-T (Bagley et al.)	Anti-EGFRvIII/anti-EGFR TEAM (Choi et al.)
Clinical trial identifier		NCT02208362	NCT05168423	NCT05660369
Study population	**Patients enrolled (n)**	92	56	3^a^
	**Patients treated (n)**	65	18	3
	**Histology**	Grade III (12%) -IV glioma	Grade IV glioma	Grade IV glioma
	**Molecular features**	IDH mutated and wild type	IDH wild type	IDH wild type
	**2**^**nd**^**-3**^**rd/n**^**relapse before CAR-T (n;%)**	43/57 (75%)	7/18 (39%)	None
	**Screening for CAR-T cell target**	IL-13Rα2 tumor expression (IHC)	EGFR amplification (FISH)	EGFRvIII expression (IHC) and EGFR amplification (routine sequencing and/or FISH)
	**Steroid limit**	6 mg	4 mg	4 mg
T cell product	**Delivery route**	Intratumor/intraventricular/dual	Intraventricular	Intraventricular
	**Targeting moiety**	E12Y-mutated IL13	Humanized scFv anti-IL-13Ra2, plus humanized scFv anti-EGFR domain (epitope 806)	ScFv anti-EGFRvIII, plus a T cell engaging anti-EGFR antibody (2 in tandem scFv, anti- εCD3 and anti-EGFR wild type)
	**Manufactory failure (n;%)**	3/92 (3.3%)	None	None
	**Manufacturing time (from leukapheresis)**	About 12 days	About 11 days	At least 7 days
	**Manufacturing process**	CD3/28 expansion of peripheral blood mononuclear cells negatively selected for CD14 and CD25; positively selected for CD62L, w/wt positive selection for CD45RA	CD3/28 +IL-15 and IL-7 stimulation of peripheral blood mononucleat cells positively selected for CD4 and CD8	CD3/28 expansion of peripheral blood mononuclear cells positively selected for CD4 and CD8
	**Phenotype of the CAR-T cell backbone**	T naive/mem (cm+stem-cell mem) or T cm	Not (yet) available	Not (yet) available
	**CD4/CD8 ratio of infused T cell product**	• About 80% CD4 for T cm • About 40% CD4 for T naive/mem	Median 3.97 (range 1.2–8.7) in the first 6 cases	Median 11.2 (range 18.8–4.6)
Study design/treatment schedule	**Number of infusions**	3 ± 1	1^d^	1^c^
	**Dose administered**	3 dose levels^h^: 10-200 × 10^6^	3 dose levels^b^: 10-50 × 10^6^	10 × 10^6^
	**Number of cohorts**	5	3^d^	4^c^
Safety	**Dose limiting toxicity**	None	One case at 2.5 × 10^7^	None
	**Grade 3–4 Adverse Events (n;%)**	23/65 (35%)	NA (33 events)	2/3 (66%)
	**Grade 5 Adverse Events (n;%)**	None	None	None
	**ICANS/TIAN**	4^e^/65 (6%)	10/18 (56%)/3/18 (17%)	3/3 (100%) G2
	**Time to Adverse Events onset p.i. (hours)**	Unknown	12–48	Fever occurred at about 24 h
	**Toxicity management**	Steroids and supportive care	Steroids and anakinra	Anakinra^f^
	**Reversibility**	2 pts reporting G4 cerebral edema (improved after steroids increase≥16 mg/day)	17/18^g^	3/3
Preliminary activity	**Objective response rate mRANO at any time point (n; %)**	4/58 (6.9%)	1/13 (8%)	3/3
	**Disease Control Rate**	29/58 (50%)	9/13 (69%)	3/3
	**Duration of tumor shrinkage**	≥90 days in 13/58 (22%)	>28 days in 8/13 (62%)	>150 days in 1/3
	**Median time to follow-up (months)**	Not available (up to 48 months)	8.1	Not available
Correlative laboratory data	**CAR-T cell persistence in CSF**	≥24 h after C1; ≥7 days in a pt subset (decrease by day 7)	Decline after 14–28 days (peak 4–10 days)	Day 3-28
	**CAR-T cell persistence in blood**	≥24 h after C1 until about 1 month after last infusion	Day 7–28 post infusion	Decline after 2–3 weeks (peak day 21)
	**CSF inflammatory cytokine levels**	≥24 h; decreasing after day 7	Decline by day 14	Decline after day 7 (but still detectable at week 4)
	**Predictive factors of activity**	• Intratumor T cell density • Increase in inflammatory cytokines in CSF and tumor cavity fluid 24hrs p.i.	Not (yet) available	Not (yet) available

a Safety run-in cohort envisioned 3 pts in case of no dose limiting toxicity within 30 days. Accrual is ongoing for 3 other arms.

b Amended dose in the updated report: 0.5–2.5 × 10^7^. Dose level 3 (5 × 10^7^) never opened for enrollment.

c In the other 3 arms patients will receive 6 weekly doses.

d Protocol amended to administer a 2^nd^ infusion on day+14.

e CAR-T attributable CNS events (G3 ataxia; G3 encephalopathy; transient G4 cerebral edema).

f None of the participants received glucocorticoids during the initial post-treatment phase.

g Back to baseline by day +28.

h Dose escalation at each cycle.

### Anti-IL13Rα2 CAR-T cell (Brown et al.)

Built up on previous experience, the improved version of the anti-IL-13Rα2 CAR-T cells described in a previous case report^41^ was tested in the largest phase I CAR-T cell trial in GMB published so far.^53^ This study included 92 patients with recurrent high-grade glioma (GMB and grade III-IV astrocytoma). Consistent with the knowledge acquired from clinical observation and progress in the field, the protocol underwent various amendments with the opening of additional treatment arms, from 2 to 5. The 5 arms were distinct in terms of timing of CAR-T cells injection (post biopsy in arm 1, post resection in arms 2-5), type of intracranial administration (intra-tumoral in arms 1–2, intraventricular in arm 3, and dual intratumoral and intraventricular in arms 4–5), and phenotype of the CAR-T cell backbone (CD62L+ CD45RA− central memory cells in arms 1–4; CD62L+ cells, comprising naive, stem-cell memory and central memory in arm 5). Across the 5 arms, CAR-T cells were administered in 3 weekly cycles plus and optional 4^th^ cycle, at 3 escalating dose levels (Table 1). IL-13Rα2 expression was evaluated with immunohistochemistry (IHC), and steroid limit was set at 6 mg/day of dexamethasone. Fifty-eight of 92 patients received three doses of CAR-T cells and were considered evaluable for safety, the primary endpoint of the study. Importantly, no dose-limiting toxicity was observed, with the most common adverse events being fatigue, headache, and hypertension. The maximum feasible dose, the other primary endpoint of the study, was defined as 200 × 10^6^. Disease control rate, assessed with the modified RANO criteria, was 50%. Three of the 4 objective responses were observed in IDH1 grade 3 gliomas. In 13 cases, stable disease or better persisted for longer than 3 months. Median overall survival (mOS) was 7.7 months; however, when considering treatment arm, a mOS of 10 versus 6 months was seen in arm 5 (14 patients) and in arms 1–4 (27 patients), respectively. This difference was significant, despite the fact that the numbers were small and the analysis was post hoc. Of note, in arm 5, the proportion of tumors with dense T cell infiltration in baseline tumor biopsy was higher and was a predictive factor of response in the global study population. An early increase in inflammatory cytokines (IFN-γ, CXCL9, and CXCL10) in the cerebrospinal fluid (CSF) and the tumor cavity fluid was seen 24 h after each infusion and was favorably associated with outcome. However, following this early increase, the level of inflammatory cytokines decreased within a week and was not strongly influenced by the number of cycles. The cytokines level change in the blood was modest. The persistence and peripheral trafficking of IL13Rα2 CAR-T cells in the CSF followed a similar pattern to that of the inflammatory cytokines, increasing as early as 24 h after CAR-T cells infusion and waning in a week. However, in a few cases, CAR-T cells were detected in the CSF for over one week. While the persistence of CAR-T cells in the CSF and tumor cavity was not influenced by the treatment arm, or effector markers expression, in the blood there was a positive association of CAR-T cell persistence with the dual delivery (arm 5), and with CD27 and LAG-3 expression on CAR-T cells. Regarding the best manufacturing protocol for the IL13Rα2 CAR-T cells, CD25 depletion after leukapheresis enabled to reduce the number of T regulatory cells in the final product. Compared with the CD62L+ CD45RA- backbone, the CD62L+ T cell (naive, central memory and stem-cell memory) used in arm 5 was superior in terms of better yield, a more favorable phenotype of the final product (lower CD57; higher CD27, CCR7, CD62L), with an improved proliferation and higher anti-tumor activity in mice models. Based on these findings, the schedule and the T cell manufacturing process used in arm 5 are under investigation in the ensuing phase II trial.

### Bivalent anti-EGFR/IL13Rα2 CAR-T cell (Bagley et al.)

To boost GBM cell killing and curb resistance from antigen loss, the first bivalent intracranial CAR-T cell was developed, with the interim data released in March 2024 on the first 6 patients and the full analysis in June 2025.^54^^,^^55^ The bivalent CAR-T cell co-expresses IL13Rα2 and EGFR-806 scFvs, where the epitope 806 of EGFR is present in EGFRvIII or EGFR overexpressing GBM (50–60%). The June 2025 report represents a clinical series of 18 patients, with a median follow-up of 8.1 months. EGFR overexpression, evaluated by fluorescent *in situ* hybridization (FISH), and recurrent GBM were key inclusion criteria. The trial envisioned 3 dose levels, initially corresponding to 1 × 10^7^, 2.5 × 10^7^, and 5 × 10^7^ CAR-T cells administered as a single dose through an intraventricular Ommaya reservoir.^54^ However, the protocol was amended after the interim analysis and the 5 × 10^7^ CAR-T cells cohort never opened, establishing the maximum tolerated dose at 2.5 × 10^7^.^55^ Regarding safety, grade 1–2 CRS and neurotoxicity were observed in all the study population. Fifty-six percent of the patients reported grade 3 early ICANS-like neurotoxicity, which was graded according to an institution-based system that accounts for baseline neurological deficits. In contrast with the *bona fide* ICANS observed in hematological malignancies after CAR-T cells infusions, the neurotoxicity was hyperacute, presenting 12–48 h post infusion. Tumor-inflammation associated neurotoxicity (TIAN) was observed in 2 of 18 patients as grade 3 and in one patient (6%) as grade 4, presenting as obstructive hydrocephalus due to peritumoral edema. Toxicity was not associated with the dose level of CAR-T cells and was managed with steroids and anakinra (anti-IL1R), or with supportive care alone. Neurotoxicity resolved within 28 days, except from one patient who presented late-onset chronic neurotoxicity, with spontaneous improvement after 9 months. Another case met the definition of dose-limiting toxicity because of prolonged fatigue, muscle weakness, and anorexia during steroids taper 2 weeks after CAR-T infusion.

According to the modified RANO criteria, there was one objective response. However, a tumor shrinkage not falling under the RANO criteria, persisting for at least 28 days, occurred in 8 of 13 cases (62%). Median PFS was 1.9 months (90% confidence interval, 1.1–3.4 months), where most patients experienced tumor progression within 1–3 months. Median OS was not reached at the time of data cut-off, with 6 of the 9 patients with at least 9 months of follow-up still alive. Following the data of the interim analysis showing early, transient tumor shrinkage as early as 24–48 h post infusion, the protocol was amended to allow a second dose after day +28 in case of clinical benefit. Of the 18 patients, 5 received this supplementary dose (28%) and had at least 1 month of follow-up.

The correlative laboratory data showed that CAR-T cells persisted in the CSF for at least 28 days, with a trend for progressive decrease after day 14 (peak day 4–10). In the blood, CAR-T cells were detected by day 7 after infusion and started to decline by day 21. CAR-T cell expansion and persistence in the CSF and in blood were positively associated with the higher dose levels (2.5 × 10^7^). Similarly to the study by Brown et al.,^53^ the peak in inflammatory cytokines in the CSF was observed at 24 h, and returned to baseline levels within 1–2 weeks.

### Anti-EGFRvIII CAR-T cell armed with a secreted anti-EGFR antibody engaging T cells (Choi et al.)

In March 2024, also the MGH group released a brief report on the 3 patients enrolled in the safety run-in cohort of the INCIPIENT trial.^56^ This study investigates an engineered T cell product of novel generation that combines a CAR-T cell targeting EGFRvIII while secreting a bivalent monoclonal antibody with a T cell-engaging and an anti-EGFR domain (TEAM). Upon engagement of EGFRvIII, the release of the T cell-engaging antibody aims at inducing the activation of endogenous T cells against EGFR expressing GBM cells.^39^ EGFRvIII status was assessed with next-generation sequencing platforms. Notably, the trial includes both recurrent and newly diagnosed GBM, even though the 3 patients in the safety run-in cohort all presented recurrent disease. The CAR-T cell/TEAM was delivered intra-ventricularly through an Ommaya reservoir as a single dose of 10 × 10^6^ cells. No dose-limiting toxicity was observed, with 2 patients experiencing grade 3 encephalopathy and fatigue, respectively. All participants presented fever after infusion, which peaked by day 2. All adverse events were managed with anakinra alone, in the absence of glucocorticoids. Following initial early regression, 2 patients experienced disease relapse within 4 weeks, while one patient reported a prolonged response, ongoing for over 3 months (last follow-up). CAR-T cells and TEAMs were transiently present in the CSF after infusion, before declining, but remaining detectable for about 4 weeks. In the blood, CAR-T cells started to increase after day 7 and peaked at day 21. Encouragingly in terms of on-target off tumor toxicity, TEAMs were found in peripheral blood only in one case, at a frequency as low as 2%. Inflammatory cytokine kinetics reflected the same pattern observed in the other 2 studies, with an early increase and return to baseline level in after about one week.^53^^,^^54^ Levels of EGFR and EGFRvIII RNA copies (measured as extra-cellular vesicle RNA) were longitudinally measured in the CSF. In participant #1, who underwent surgical resection after disease progression, the resected tumor still moderately expressed EGFR, while EGFRvIII was absent. In participant #3, the GBM re-biopsy before CAR-T cell/TEAM had lost EGFRvIII expression, initially present at diagnosis, while EGFR expression was preserved.

### Cross-trial considerations

Detailed information on the three studies are reported in Table 1. Despite the obvious differences in terms of product, type of target(s), study population (the study by Brown et al. included also IDH+ grade III-IV gliomas), and schedule of administration, the trials share considerable commonalities.

Even though radiological responses were transient, they occurred as early as 24 h after infusion, at least in study led by Bagley^54^ and that by Choi,^56^ while in Brown et al.^53^ the first radiological imaging available was at one month. In most cases, the early tumor shrinkage was followed by a dimensional increase falling under the criteria of disease progression according to the RANO/modified RANO criteria. However, in at least 2 cases in the bivalent CAR-T cell report,^54^ after initial shrinkage, the progressing tumor lesion was still smaller compared with the baseline dimensions and remained stable or even decreased over time. Additionally, 15 patients in the IL-13Rα2 trial were alive at 12 months after accrual, despite radiological disease progression.^53^ Similarly, in the bivalent CAR-T cell trial, at least 6 of 18 patients had an OS longer than 9 months (immature follow-up).^55^ It will be important to assess if prolonged post-progression survival can be observed on a larger population treated with CAR-T cells. Similarly to immune-related RECIST, in immunotherapy RANO criteria^57^ “pseudoprogression” is taken in consideration, proposing a confirmatory MRI scan at 12 weeks. These criteria were not considered for response evaluation in the study by Brown et al.^53^ or in that from Bagley et al.,^54^ while their integration is mentioned in the protocol of the INCIPIENT trial, even though their use is unclear in the reports of the safety run-in cohort.^56^ It should be considered that immunotherapy RANO criteria were primary developed to assess response to immune checkpoint inhibitors. As more patients will be treated with CAR-T cell therapy, differences in the response kinetics from checkpoint inhibitors will become more evident, likely leading to the amendment of these criteria and their use for response evaluation to engineered T cell therapies.

The timing of occurrence of radiological response coincided with the presentation of treatment-related toxicity and the peak of the inflammatory cytokines in the CSF. This highlights how difficult it can be to uncouple the toxicity and the anti-tumor response of CAR-T cell therapy, as they are both linked to inflammation. Along the same lines, the administration of immune-suppressive treatments on the one hand mitigates the life-threatening adverse event, while on the other it may attenuate the anti-tumor efficacy of CAR-T cells.

Compared with CAR-T cell therapies in lymphoproliferative disease, in GBM, moderate/severe CRS was absent. Potential causes could be low antigen binding, faster CAR-T cell exhaustion, and the higher burden and distribution of the antigens targeted by the CAR-T cells in hematology (e.g., CD19). Transient neurological symptoms were experienced by the large majority of patients. In this context it can be difficult to determine the cause of neurological symptoms or neurological deterioration. Since in hematological patients ICANS is strongly associated with higher-grade CRS, it is possible that the neurotoxicity observed in the GBM CAR-T cell trials is more likely TIAN, with increased intra-cranial pressure and edema resulting from local inflammation, at the level of tumor, neural, and glial cells.^58^ The appropriate recognition of the mechanisms and components of neurotoxicity in this context is relevant, with implications on future treatment approaches to selectively mitigate toxicity while avoiding immune-suppression.

In all the three studies,^53^^,^^54^^,^^56^ CAR-T cells persisted longer in the blood than in the CSF, consistent with the poor T cell survival upon exposure to the chronic inflammatory signals and the hypoxic conditions in GBM. To address this issue, the development of engineered T cells able to withstand the immune inhibitory milieu is a current research priority, as further discussed in section “perspectives to improve CAR-T cell homing at the tumor site.”

Encouragingly, the manufacturing failure rate was low in all the three trials,^53^^,^^54^^,^^56^ and the manufacturing time was shorter than 2 weeks, as leukapheresis was performed in advance. This improved time efficiency reflects the technological progress and the experience acquired by treating hematological malignancies. Importantly, the reduced time of production demonstrates that CAR-T cell therapy can be feasible in routine clinical practice on a larger scale, in contrast to what was previously believed.

Certainly, data on the activity of these loco-regionally administered CAR-T cells, especially for the CAR-T cell/TEAM, are immature. A higher number of patients treated and longer follow-up data will help to address the open questions discussed here.

## Perspectives to improve CAR-T cell homing at the tumor site

**Figure 1:**
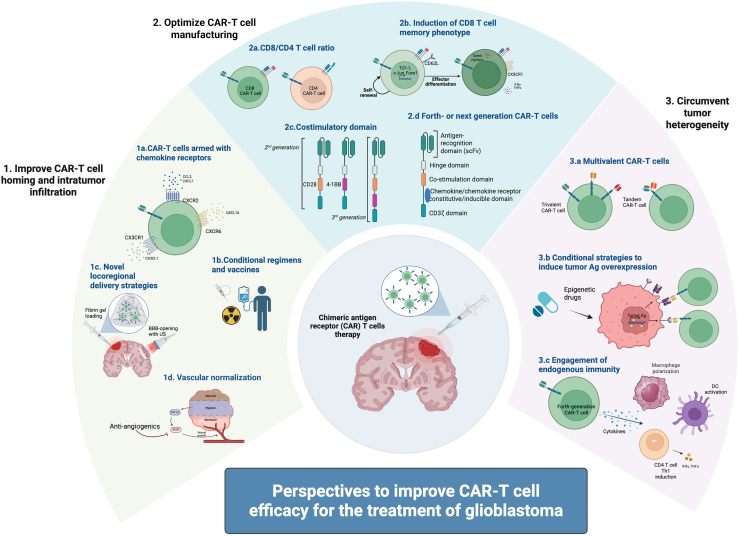
Perspectives to improve CAR-T cell efficacy for the treatment of glioblastoma Created in https://BioRender.com.

The translational findings in all the three studies and the previous pilots summarized above indicate that CAR-T cells have limited persistence at the tumor site. To circumvent this major issue for the use of CAR-T cell in solid tumors, several approaches are under investigation, detailed below and summarized in Figure 1.

### CAR-T cells armed with chemokine receptors

CAR-T cells expressing chemokine receptors that match the chemokines produced in the tumor microenvironment were proposed already 15 years ago, in the attempt to increase CAR-T cell homing and intratumor persistence.^59^ A preclinical study assessed the intratumor penetration and viability of anti-CD30 CAR-T cells expressing CCR4 using *in vivo* imaging in a severe combined immunodeficiency mice bearing subcutaneous Hodgkin lymphoma. CCR4 is a receptor ligating CCL22 and CCL17 chemokines, produced by intra-tumor macrophages and tumor cells. While CCR4 is absent on effector CD8 T cells, it is present on T helper 2 and regulatory T cells, which further contribute to the immune-suppressive microenvironment of Hodgkin lymphoma. In this model, CCR4-expressing CAR-T cells showed improved *in vitro* and *in vivo* migration, and anti-tumor control compared to CCR4-negative CAR-T cells.^59^ As another example, in GBM murine xenografts, CXCR1 and CXCR2-expressing anti-CD-70 CAR-T cells showed increased intratumor accumulation early after administration, with longer intratumor persistence. This eventually resulted in improved anti-tumor activity and long-lasting immunologic memory at tumor rechallenge. In this experiment, mice were pre-conditioned with radiation therapy, to increase the expression of the cognate ligands CCL2, CCL20, and CXCL1, 2, and 8.^60^ Among others, interesting chemokine receptors tested in animal models to increase chemotaxis of endogenous T cells are CXCR6^61^ and CX3CR1.^62^ As these chemokine receptors are also well-established markers of effector CD8 T cells in cancer and in chronic infection,^63^^,^^64^^,^^65^ it is possible that engineered T cell overexpressing the CX3CR1 or CXCR6 receptors may not only display improved homing, but also enhance effector functions, to be confirmed in further studies. Of note, engineered T cells expressing CXCR2 and CCR4 have entered clinical experimentation. The NCT01740557 trial, testing CXCR2-transduced autologous tumor infiltrating lymphocytes in melanoma, was completed in 2023, with results not yet available. Another phase I trial (NCT01740557) is recruiting patients with multiple myeloma to be treated with a CCR4-expressing anti-CD30 CAR-T cell. In primary or metastatic brain tumors, a sequestration of circulating T cells in the bone marrow has been described.^66^ This was shown to depends on cancer signals inducing a loss of the sphingosine-1-phosphate receptor 1 on T cells, a regulator of T cell egress from lymphoid tissues. Blockade of the internalization of this receptor licensed T cell trafficking to the brain and improved the activity of double checkpoint inhibitors (4-1BB and PD-1) in murine models of GBM. This phenomenon should be taken into account when developing T cell adoptive therapies for brain tumors and suggests that engineering CAR-T cells to express the sphingosine-1-phosphate receptor 1 could be advantageous to avoid CAR-T cells and activated endogenous T cells in the bone marrow.

### Conditional regimens and vaccines

The penetration and intratumor persistence of CAR-T cells is enhanced by direct targeting and elimination of the immune-suppressive myeloid cells that are abundant in GBM. These myeloid cells suppresses T cell cytotoxic functions by cytokine production (e.g., IL-10, IL-1, TGF-β) and by contributing to an hypoxic and acidic milieu. Reprogramming of immune-suppressive macrophages is a difficult endeavor, mostly for the pleiotropic activity and redundancy of the chemokines and cytokines they sense and produce.^67^^,^^68^ CAR-T cells that target specific subsets of macrophages expressing the folate receptor β were tested in immune-competent murine models of ovarian cancer, colon cancer, and melanoma.^69^ Conditional treatment with an anti-folate receptor β CAR-T cells significantly improved the anti-tumor efficacy of later-infused anti-mesothelin CAR-T cells. This was associated with an enrichment of pro-inflammatory monocytes and with a higher number and frequency of endogenous tumor-specific CD8 T cells at the tumor site. Interestingly, when the anti-folate receptor β CAR-T cells and anti-mesothelin CAR-T cells were given concomitantly, the anti-tumor efficacy was abrogated. In an immune-competent GBM mouse model, the re-polarization of tumor-infiltrating myeloid cells toward a pro-inflammatory, T cell-supportive state was obtained with the implant of a device in the surgical cavity that slowly releases small molecules inducing IL-12 production and T cell infiltration.^70^

Another preclinical study investigated CAR-T cells targeting the anti-macrophage marker F4/80^71^ in an immune-competent orthotopic model of lung cancer. The IFN-γ secreted by CAR-T cells upon antigen (F4/80) binding increased antigen presentation and expansion of endogenous tumor-specific CD8 T cells, ultimately delaying tumor growth.

One more perspective is to deplete the extracellular matrix of the tumor microenvironment, which is particularly attractive for tumors with an intense stromal component (i.e., pancreatic adenocarcinoma, GBM, mesothelioma). In mice models, CAR-T cells targeting the fibroblast activation protein (FAP) rapidly depleted matrix, enhanced T cell infiltration, and promoted the adaptive immunity and the expansion of anti-mesothelin CAR-T cells, which were administered subsequently.^72^ Notably, the results of a small phase I pilot trial of intrapleural injection of FAP-targeting CAR-T cells proved safe and feasible in three patients affected by pleural mesothelioma.^73^

To foster the penetration and persistence of CAR-T cells in GBM, a less specific but more practical approach consists of conditional cytotoxic treatments, like chemotherapy, as already in use for CAR-T cells in hematologic malignancies.^74^ Conditional cytotoxic therapy presents multiple advantages. Not only does it reduce tumor burden, reducing hypoxia and improving the penetration of CAR-T cells, but it is also able to deplete pro-tumoral myeloid cells, T regulatory cells, and endogenous T cells terminally exhausted.^75^ In acute leukemia, conditional lymphodepleting chemotherapy has been associated with better clinical response and longer persistence of CD4^+^ and CD8^+^ CAR-T cells.^76^ A further advantage of conditional chemotherapy is that CAR-T cells infused during the ensuing lymphopenic phase would undergo homeostatic proliferation, which is mediated by the physiological increase of IL-15, IL-7, and IL-21, produced by the bone marrow to replenish the immune system.^75^^,^^77^^,^^78^ Even though this approach failed to improve persistence and activity of intravenous CAR-T cells in GBM,^43^^,^^44^ how conditional cytotoxic treatment impacts the persistence and function of locally administered CAR-T cells in GBM is still unknown.^56^

A promising approach promoting the intratumor migration and local expansion of CAR-T cells is their combination with CAR-T cells-amplifying vaccines. In a recent phase I trial in relapsed or refractory solid tumor,^79^ the safety and preliminary activity of CAR-T cells targeting claudin-6 was assessed with and without the concomitant administration of an mRNA vaccine encoding for the cognate target antigen, claudin-6. In preclinical models, the administration of the vaccine facilitates systemic antigen delivery and presentation to antigen-presenting cells in lymphoid organs, mediating *in vivo* stimulation and controlled expansion of CAR-T cells, resulting in tumor control even at subtherapeutic CAR-T cell doses.^80^ In the clinical trial, 19 of 22 patients reported grade≥3 toxicities, including 2 dose limiting toxicities (hemophagocytic lymphohistiocytosis and prolonged pancytopenia), both resolved. Overall response rate was observed in 33% of the patients, with partial responses deepening over time.^79^ This trial in ongoing with final results eagerly awaited.

### Novel technologies and devices to improve loco-regional delivery and intratumor penetration

Based on the preliminary data reviewed here, the loco-regional administration of CAR-T cells in the intraventricular space through the Ommaya reservoir enhances their penetration in GBM compared with the peripheral infusion and presents the further advantage of a lower systemic toxicity. Novel evidence suggests that the loco-regional delivery of CAR-T cells could be further optimized.^81^ A recent study showed that encapsulating CAR-T cells in a fibrin gel promoted their gradual local release, sustaining CAR-T cell viability and prolonging their anti-tumor activity, compared with CAR-T cells directly inoculated in the tumor resection cavity of a murine GBM model.^82^ The intraparenchymal delivery through convection-enhanced devices, known as Renishaw, could be an alternative to the Ommaya reservoir.^83^ The technique envisions the implantation of catheters through which conventional and novel therapeutic formulations can be delivered using continuous, low-positive-pressure bulk flow, with the expected major complication being fluid backflow. So far, there is no experience of intraparenchymal convection-enhanced administration of CAR-T cells, and the available evidence in high-grade gliomas remains limited, yielding disappointing results with the infusion of chemotherapy.^84^^,^^85^

A different device-based strategy seeks to overcome the BBB by disrupting it, thereby improving the delivery of systemic drugs to the tumor site, potentially including CAR-T cells. The most promising is an implantable device delivering low-intensity pulsed ultrasound with concomitant intravenous microbubbles, to transiently increase BBB permeability in targeted areas of the CNS.^86^^,^^87^ A recent phase I/II multicenter clinical trial evaluated the safety of a nine-emitter implantable device, SonoCloud-9, and its efficacy at BBB opening in patients with recurrent GBM.^88^ The device was activated before or after carboplatin infusion every 4 weeks, and showed a BBB opening for about 1 h. No dose-limiting toxicities were observed, with the most common adverse event being pain at the time of the device connection. Based on these results, SonoCloud-9 in combination with carboplatin is under investigation in a phase III trial, compared with chemotherapy alone, in recurrent GBM (NCT05902169). An earlier phase trial is testing this approach in newly diagnosed GBM (NCT04614493). Laser interstitial thermal therapy is an alternative technique to disrupt the BBB, based on a laser probe placed intracranially under a magnetic resonance imaging guide.^81^ Compared with the ultrasound-based device, the laser-based opening of BBB appears more invasive and less manageable in terms of side effects. As an ablative therapy for intracranial tumors, laser interstitial thermal therapy has been approved by the Food and Drug Administration, but its potential as an induction strategy to improve the intratumor penetration of drugs is under evaluation in early clinical trials in combination with PD-1 blockade or chemotherapy.^81^^,^^89^^,^^90^^,^^91^ Electroporation and electrical fields represent further potential device-assisted technologies to disrupt the BBB to improve drug delivery.^81^ Despite the absence of active trials combining these devices with CAR-T cells in GBM, the idea of a transient BBB permeabilization, through ultrasound or electroporation, prior to systemic administration of CAR-T cells would represent an intriguing perspective to increase intratumor penetration.

### Vascular normalization of the tumor microenvironment

Vascular normalization is another key concept to improve intratumor penetration of CAR-T cells.^92^ Thanks to novel techniques of single-cell RNA sequencing, an atlas of the brain vasculature has been recently described, across health, fetal development, and a variety of diseases, including GBM.^93^ So far, the results are non-specific concerning GBM, but they overall confirm neo-angiogenesis and suggest stem-to-endothelial cell transdifferentiation. A signature of BBB dysfunction and lower MHC class II expression was also noticed. Another work carried out a transcriptional and spatial profiling of mural and endothelial cells across primary and metastatic brain tumors.^94^As expected, the most extensive changes in structural and molecular vasculature, compared with non-tumor brain tissue, were seen in GBM and brain metastases, with the latter distinctively presenting interferon-stimulated gene signature and upregulation of signals suggesting immune cells interaction.

Although the concept of vessel normalization is intriguing, its pursuit remains challenging. This difficulty stems from the lack of predictive biomarkers and quantitative methods to evaluate tumor vascular networks. Indeed vascularization is a dynamic process upon continuous vessel pruning and resulting hypoxia.^92^ In a preclinical orthotopic GBM model, tumor vasculature and oxygenation statuses were assessed using fluorescence microscopic imaging and deep learning-based quantitative analysis.^95^ A combination of chemotherapy and bevacizumab induced vasculature normalization, and chemotherapy alone reduced hypoxia, probably for an indirect effect due to reduced tumor burden. The interpretation of these data is limited by the use of an immune-deficient mouse model but indicates than an improved understanding of vascular normalization is still required.

## Perspectives to improve CAR-T cell construct and effector functions

### Role of CD4/CD8 T cell proportion and co-stimulatory domain

Once CAR-T cells make their way into the tumor bed, their ability to expand and survive in the tumor microenvironment is paramount for sustained anti-tumor control. This capacity of CAR-T cells primarily depends on the manufacturing process (Figure 1). First, the final composition of the CAR-T cell product in terms of CD4/CD8 ratio is important for their function and therapeutic efficacy. Dedicated studies have shown synergy between CD4^+^ and CD8^+^ CAR-T cells,^76^^,^^96^ showing that a 1:1 ratio of CD4:CD8 has improved potency compared to unselected T cells. During the manufacturing process, stimulation and co-stimulation of CAR-T cells also play a critical role. In preclinical studies and retrospective analyses, second/third-generation 4-1BB+ CAR-T cells showed longer persistence and lower toxicity compared with CD28^+^ CAR-T cells.^97^ However, this comparison is limited by the lack of prospective randomized data and the fact that the CAR-T cells tested in clinical trials differ in other aspects aside from the type of costimulatory domain.^34^

### Induction of a CD8 T cell memory phenotype in CAR-T cells

The phenotype of the starting T cell has a major role in determining the expansion potential and long-term persistence of the final CAR-T cell product (Figure 1). In hematological malignancies, (pre-infusion) CAR-T cells from patients achieving a complete response were enriched in memory-related genes.^98^^,^^99^^,^^100^ Inducing memory functions in CAR-T cells is a of utmost importance in the field of T cell engineering. Upon re-exposure to the cognate antigen, memory T cells have the capacity to rapidly differentiate into effector cells while continuing to self-renew, thus providing rapid antigen clearance and long-term protection.^101^^,^^102^ Enhancing the memory phenotype of CAR-T cells offers additional benefits in terms of reduced yield requirements: a rapid self-renewal and effector burst at the tumor site may be more critical than the absolute dose of CAR-T cells infused. Within the CD8 T cell memory subset, it is well established that CAR-T cells isolated from central memory T cells are superior to those deriving from effector memory cells, which are probably already exhausted at the time of infusion after *in vitro* culture, with poor expansion potential.^96^^,^^103^^,^^104^ Even more so, as shown in the trial by Brown^53^ and by others,^105^^,^^106^ the CD8 T cell stem-cell/memory phenotype seems to be the best for CAR-T cell activity.^96^ This subset is thought to be the least differentiated and the earliest progenitor of memory T cells. As such, it is endowed with an increased proliferative capacity and a faster differentiation, compared with T memory cells as a whole. Similarly to naive T cells, stem-like memory T cells are CD45RA positive and CD62L/CCR7 positive; however, they differ in the expression of CD95 (FAS), CD122, CXCR3, and LFA-1.^107^

Since this subset is present at very low frequency in the blood, and thus it is difficult to enrich after leukapheresis for CAR-T cells, multiple strategies are under study to induce a stem-cell memory CD8 T cell phenotype *in vitro*, during the manufacturing process. Among those, small molecules mostly activating the *Wnt* beta-catenin pathway^108^ during *in vitro* culture and expansion of CAR-T cells improved the function and survival of the final T cell product^109^^,^^110^^,^^111^^,^^112^ in animal models and are currently being investigated in clinical trials (NCT06500819; NCT04196413). Genetic manipulations with the synthetic overexpression of key transcription factors involved in memory formation, like *FOXO-1* or *c-Jun,* has also been found to enforce differentiation toward an early memory phenotype in CAR-T cells. In tumor-bearing mice overexpression of these transcription factors reflected in enhanced persistence and slower exhaustion of the modified CAR-T cells.^113^^,^^114^ Alternatively, a stem-like/memory phenotype is induced through metabolic changes, modulating the differentiation fate of T cells at the epigenetic level.^115^^,^^116^ Compared with effector T cells, memory T cells rely more on mitochondrial metabolism and oxidative phosphorylation.^117^ Metabolic reprogramming with the inhibition of the mitochondrial pyruvate carrier robustly induced a stem cell-like memory phenotype in CAR-T cells from healthy donors and patients. Improved CAR-T cell effector functions in mouse models were achieved also through manipulation of glutamine reductive carboxylation, where the genetic deletion of this enzyme in CAR-T cells led to improved effector function and antitumor activity.^118^ The metabolic requirements of CAR-T cells can be regulated by certain cytokines as well. Stimulation with IL-15 and IL-7 has already shown to induce an immature, pluripotent stem-cell memory phenotype in cultured T cells for CAR-T cell manufacturing.^119^^,^^120^^,^^121^ CAR-T cell trials testing IL-15 in generating less-differentiated pluripotent CAR-T cells in solid tumors are ongoing (NCT05103631, NCT04715191, NCT04377932, NCT03721068).

It has been proposed that the induction of a memory phenotype is beneficial for hematological malignancies, whereas for the use of CAR-T cells in solid tumor inducing a memory-like phenotype could be counterproductive.^122^ This consideration is based on the fact that memory/naive T cells, expressing the CD62L/CCR7 receptors, will migrate to lymphoid organs and they will not survive in the hostile tumor microenvironment. However, the desirable final outcome of using this phenotype (at the time of infusion) is that these stem-like/memory cells once infused in the patients will differentiate in effectors and be able to penetrate and survive in solid tumors long enough to eliminate the cancer cells expressing their cognate antigen. Meanwhile, to provide immunological memory, these cells will still slowly self-renew establishing a niche in lymphoid tissue, or, if developing a resident memory program, even within tumors.^123^

### Importance of CD4 T cell characteristics in the CAR-T cell pool

It has become increasingly recognized that in addition to the CD8 T cell memory phenotype CD4 T cell characteristics matter for the *in vivo* longevity of the final pool of CAR-T cells. Single-cell RNA sequencing analysis revealed that pre-infusion (unstimulated) anti-CD19 CAR-T cells in patients with ultra long remission from leukemia were transcriptionally enriched in memory-associated clusters characterized by markers such as *CD27*, *TCF7*, *CCR7*, and *IL7R*. In contrast, CAR-T cells from relapsed patients exhibited reduced enrichment in these clusters.^100^^,^^124^ After re-stimulation with antigen-presenting cells, these pre-treatment CAR-T cells from long responders were still enriched in the memory clusters (consistent with the self-renewal capacity of memory cells), but they were also enriched in a small cluster showing type 2 functionality. This cluster was composed predominantly of CD4 T cells overexpressing Th2 cytokines (*IL4, IL5*) and the transcription factor *GATA3*. Using *in vitro* and *in vivo* functional experiments, IL-4 exposure of CAR-T cells is able to improve longevity, and effector differentiation of CAR-T cells, ultimately resulting in improved tumor control.^100^^,^^124^ A co-published work in tumor-bearing mice similarly proposes that an IL-4 fusion protein improves the effector functions of terminally differentiated CD8 T cells by increasing glycolytic metabolism in terminally effector cells through activation of the STAT6 and mTOR pathways.^125^ However, as previous data showed that IL-4 exposure facilitates CD8 T cell exhaustion,^126^ further mechanistic data will be needed to better clarify the causal relationship between type 2 signals and CAR-T cells functionality.

### Hypoxia-resilient CAR-T cells

The acidic microenvironment resulting from the disruptive vascularization and hypoxia is known to hamper the effector functions and the intratumor persistence of CD8 T cells.^127^^,^^128^ Antigen-specific CD8 T cells show reduced cytokine secretion (IFN-γ, IL-2, and TNF) and proliferation at low pH, because of a reduced responsiveness to autocrine IL-2 and decreased mTORC1 signaling activity. Engineering CD8 T cells with increased mTORC1 did not improve T cell functions in acidic conditions indicating that further factors may be at play.^129^ However, *in vitro* exposure of CAR-T cells to the AMPK activator metformin and the mTOR inhibitor rapamycin, during manufacturing, generated products with durable and effective anti-glioma cytotoxic activity under hypoxic conditions in a mouse model.^130^ In the same study, preconditioning of human CAR-T cells similarly improved cytotoxic functions under *in vitro* hypoxic condition.

### New synthetic T cell constructs

Over the past few years, T cell engineering has evolved into the development of a variety of complex T cell constructs in addition to the CAR generation.

The most notable example is CAR-T cells endowed with autocrine cytokine stimulation. The third signal of the immune synapsis is artificially provided by the CAR-T cell itself, with the aim of amplifying the cytotoxic response, while sustaining its own survival. These fourth-generation CAR-T cells are known as T cells re-directed for universal (antigen-unrestricted) cytokine-initiated killing (TRUCKs).^131^^,^^132^ Several TRUCKs have been developed and tested in early phase clinical trials. These vary according to the cytokine secreted upon activation, which is chosen based on the desired outcome. To enhance the potency and persistence of CAR-T cells *in vivo,* TRUCKs have been armed with IL-2, IL-15, IL-7, IL-21, and IL-23.^132^^,^^133^ Among those, IL-15 (NCT04377932, NCT05103631) TRUCKs are currently being tested in phase I trials for the treatment of pediatric solid tumors, with an encouraging ORR of 66% in a recent preliminary report.^134^ One more example are TRUCKs that upon activation secrete IL-7 and CCL19, aiming at improving tumor trafficking and the recruitment of endogenous and synthetic T cells.^132^ In fact, CCL19 is a chemokine produced by the fibroblastic reticular cells present in the T cell zone of the lymphoid organs.^135^ These IL-7+CCL19 TRUCKs are being evaluated in phase I trials, both for the treatment of hematologic malignancies (NCT03778346 in multiple myeloma, NCT03929107, NCT04833504 in lymphoma) and solid tumors (NCT03198546).^136^^,^^137^

Beyond CARs, off-the-shelf TCR-engineered T cells are also of interest for targeting both intracellular and extracellular antigens, for superior antigen sensitivity and for reduced tonic signaling compared with CAR-T cells. A proof of principle has been recently proposed for anti-PTPRZ1 synthetic T cells, isolated from GBM patients vaccinated with PTPRZ1, an HLA-A∗02-restricted epitope associated with GBM cell stemness. Even though these TCR-engineered T cells could potentially broaden the repertoire of targetable GBM-associated antigens, they are encumbered by MHC restriction.^138^ A more sophisticated variant combining TCR and CAR engineering is synthetic chimeric TCR therapy. While still reproducing more physiologically the intracellular signaling domain of a native TCR,^139^ this construct presents a modified extracellular domain of the receptor, defined as a full chimeric TCR. This consists of an scFv fused to the TCR constant alpha chain while the TCR constant beta chain is left void. Functionally, this hybrid TCR construct showed improved sensitivity to lower antigen levels and has been readily adapted for bispecific targeting, adding one more scFv on the void TCR constant beta chain. A similar approach to induce more controlled and physiological T cell responses is that conceived for synthetic T cells with a T cell antigen coupler. This chimeric receptor co-opts the endogenous TCR, mimicking a CD3 co-receptor. Using an antigen binding moiety (i.e. ScFv), it redirects the CD3-TCR complex to a target antigen of choiceby. In terms of anti-tumor function, it showed more efficient anti-tumor responses and reduced toxicity *in vitro* and in xenograft models.^140^

Lastly, one of the most interesting perspective is the development of T cells with synthetic Notch (synNotch) receptors that sense an extracellular antigen and respond by inducing a transcriptional response.^141^ These synNotch-CAR circuits function as Boolean “AND” or ““IF-THEN” gates, requiring the recognition of both priming (synNotch) and killing (CAR) antigens. As usually the CARs are locally induced and expressed on the cell surface only upon the priming antigen binding, the advantages of this new construct are not only reduced tonic CAR intracellular signaling and exhaustion, but also increased safety and multi-antigen targeting, as further discussed in the following section.

## Perspectives to circumvent tumor heterogeneity and immune escape

Tumor heterogeneity and adaptive immune escape remain the most significant obstacles to successful targeted therapy in oncology. In the context of adoptive T cell therapy, adaptive immune escape of tumor has been referred to as “the elephant in the room,” highlighting its pervasive yet unresolved nature.^142^ As graphically summarized in Figure 1, to address tumor heterogeneity and prevent immune evasion with CAR-T cells, two majors approaches have been studied: 1) targeting of multiple tumor-specific antigens; 2) optimal stimulation of the endogenous immune system, both in terms of local bystander effect and adaptive effector response against unknown tumor-specific targets.

The first approach to target multiple tumor antigens involved the use of CAR-T cell cocktails, which consist of infusing different CAR-T cells directed against distinct antigens. These cells were administered either simultaneously or sequentially, yielding overall positive results in clinical experience.^143^^,^^144^^,^^145^ Compared with CAR-T cell cocktails, bispecific CAR-T cells envision a more sophisticated engineering but are more convenient in terms of procedure (lower yield and single infusion). Dual CAR-T cells carry two CARs signaling separately, as in the case of the bivalent anti-EGFR/IL13Ra2 discussed above.^54^ Tandem CARs represent a relatively novel design, fusing two tumor antigen-specific scFvs with one intracellular signaling moiety. Tandem CAR T cells recognize each antigen individually and enable synergistic activation when both scFvs are simultaneously engaged, with advantages on longer persistence, slower exhaustion, and antigen escape.^146^ In preclinical GBM models, both tandem divalent (anti-IL13Rα2 and -EphA2)^146^^,^^147^ and tri-targeting CAR-T cells (anti-HER/IL13Rα2/EphA2) using 3 different CARs showed positive results.^148^ Results from the ongoing phase I trial of loco-regionally infused CAR-T cells simultaneously targeting B7-H3, EGFR806, HER2, and IL13Ra2 in pediatric gliomas (NCT05768880) will provide useful information on the feasibility and preliminary activity of multi-target CAR-T cells in patients.

To reduce tumor heterogeneity, inducing the overexpression of the targeted antigen is an additional strategy under investigation, especially in hematologic tumors.^149^ In solid tumors, preliminary evidence indicates that epigenetic drugs can modulate antigen expression. For example, decitabine has been reported to enhance the expression of mucin-1 in pancreatic cancer, while histone deacetylases inhibitors can increase the expression of GD2 expression in neuroblastoma.^150^^,^^151^ Similarly, combination of CAR-T cell therapy with other conventional treatments like radiation therapy or chemotherapy has been attempted to modulate the expression and presentation of desired tumor antigens.^149^ However, so far, the combination of RT and PD-1 blockade with CAR-T cell therapy has yielded negative results in GBM.^44^

As mentioned above, an alternative bioengineering strategy to circumvent the issue of tumor antigen heterogeneity and immune escape, is the use of synNotch CAR-T cells. By screening a set of CNS-specific extracellular ligands, Simic et al. showed that brain-sensing synthetic T cells inducing CAR expression were able to treat primary and secondary brain cancers in mouse models, without off-target attack of tissues outside of the brain.^152^

The engagement of the endogenous immunity allows to expand the range of tumor cells targeted, surpassing the limitations of cognate antigen expression. Bystander killing effect on antigen negative tumor cells has already been observed in second-generation CAR-T cells targeting the GBM-associated antigen PTPRZ1, where this effect was mediated by the pro-inflammatory Th1 cytokines secreted by CAR-T cells upon antigen binding.^153^ Fourth-generation CAR-T cells and in particular TRUCKs that synthetically secrete specific cytokines or other soluble signals upon antigen binding will thereby be able to further and more specifically stimulate the host innate immune response, enhancing bystander anti-tumor immunity.^132^ IL-18, a potent inflammatory cytokine, is one of the most promising cytokine to be exploited for TRUCKs.^154^ IL-18 activates natural killers, macrophages, and T cells, which in turn would produce IFN-γ, with further recruitment and activation of more immune cells, eventually synergizing for tumor control.^155^ TRUCKs armed with a payload of non-cytokine proteins, such as membrane receptors, antibodies, enzymes, and transcriptional factors represent another variant of 4^th^-generation CAR-T cells serving the purpose of circumventing tumor antigen heterogeneity and adaptive antigen escape.^132^ The EGFRvIII/anti-EGFR TEAM of Choi et al. discussed here is a notable example that arrived at the clinical experimentation. The final results of this trial will offer important insights for further development of this type of TRUCKs in solid tumors.^56^

Beyond boosting the expansion and the intratumor effector functions of CAR-T cells (discussed in section “conditional regimens and vaccines”), vaccines encoding for the target antigen have been shown to stimulate the host innate immunity, inducing antigen spreading and priming of new tumor-specific antigens in mice models.^156^ In particular, this process seems to depend on the IFN-γ produced by the CAR-T cells, which promote the recruitment of IL-12+ dendritic cells and engage in a positive feedback loop. Indeed, through IFN-γ, CAR-T cells may be capable of polarizing the myeloid populations toward a favorable anti-tumor phenotype. After treatment with vaccine-boosted CAR T cells, complete responses in mice were seen in antigen-heterogeneous tumor, where only 50% of the cells expressed the target antigen. Taking advantage of a similar mechanism, synthetic dendritic cell progenitors secreting IL-12 and FLT3L were shown to sustain and synergize with anti-GD2 CAR-T cell therapy in a murine GBM model, potentially through engagement of endogenous immunity.^157^^,^^158^

These data highlight the importance of the crosstalk of CAR-T cells with the host immunity in determining anti-tumor responses. For the advancement of CAR-T cells to treat solid tumors, and in particular GBM, translational investigation should further clarify the optimal balance for delivering inflammatory stimuli and for eliciting an endogenous bystander effect, to avoid adaptive resistance (e.g., chronic inflammation with PD-L1 upregulation, reactive fibrosis) and toxicity.

## Metrics of success to wide acceptance from the neuro-oncology community

The path to successfully treating GBM with CAR-T cells remains long and challenging. However, the early-phase clinical trials reviewed here have yielded useful preliminary results, demonstrating the feasibility of CAR-T cell therapy in terms of manufacturing, intracranial administration, and overall safety. Moreover, even though tumor shrinkage was transient in most cases, the rare long-term responses and survival benefit are a significant finding given the grim prognosis of GBM and the scarcity of effective treatment options. Importantly, the ensuing phase II trials of the anti-IL13Rα2 CAR-T cell, and of the anti-EGFR/ IL13Ra2 CAR-T cell will also provide key information with the formal assessment of anti-tumor activity. At the same time, future updates of the phase I trial testing the EGFRvIII/EGFR-TEAM CAR-T cells—with the accrual of additional patients—will clarify many open questions, including the tumor antigen evolution upon treatment. Indeed, the translational correlative data from clinical trials will be crucial to better understand the anti-tumor mechanisms in responding patients. This will be important for the identification of predictive factors of response and for the development of treatment strategies to effectively target resistance mechanisms and to ultimately obtain sustained tumor shrinkage over time. To this aim, the recent availability of spatial transcriptomics and proteomics at the single-cell resolution will be a critical tool for tumor antigen discovery and immune dynamics.

Overall, a reason to be optimistic for the future of CAR-T cells in GBM is the accelerated progress in T cell engineering, with tunable CAR-T cells on the horizon. The next generation of CAR-T cells focuses on dynamically modulating the expression of specific proteins. This modulation is tailored to factors such as the cellular location, the time elapsed after administration, and the dynamics of antigen burden. These new technologies will be able to optimize therapeutic efficacy and minimize off-target effects of engineered T cells for the treatment of poorly immunogenic solid tumors.^132^

## Acknowledgments

The authors received no funding for this work. All figures were generated with Biorender (https://BioRender.com).

## Declaration of interests

D.M. is an inventor of patents related to CAR-T cell therapy, filed by the University of Pennsylvania, the Istituto Oncologico della Svizzera Italiana (IOSI), and the University of Geneva. D.M. is a consultant for Limula SA and MPC Therapeutics SA. D.M. is the scientific co-founder and has an equity interest in Cellula Therapeutics SA.

A.M. received financial support to attend scientific meetings for registration, travel, and accommodation from Johnson & Johnson, Roche, and PharmaMar. A.M. provided advisory services to BMS.
